# Nuclear Progesterone Receptors Are Up-Regulated by Estrogens in Neurons and Radial Glial Progenitors in the Brain of Zebrafish

**DOI:** 10.1371/journal.pone.0028375

**Published:** 2011-11-30

**Authors:** Nicolas Diotel, Arianna Servili, Marie-Madeleine Gueguen, Svetlana Mironov, Elisabeth Pellegrini, Colette Vaillant, Yong Zhu, Olivier Kah, Isabelle Anglade

**Affiliations:** 1 Neurogenesis and Oestrogens, UMR CNRS 6026, IFR140, Université de Rennes 1, Rennes, France; 2 Department of Biology, East Carolina University, Greenville, North Carolina, United States of America; Ecole Normale Supérieure de Lyon, France

## Abstract

In rodents, there is increasing evidence that nuclear progesterone receptors are transiently expressed in many regions of the developing brain, notably outside the hypothalamus. This suggests that progesterone and/or its metabolites could be involved in functions not related to reproduction, particularly in neurodevelopment. In this context, the adult fish brain is of particular interest, as it exhibits constant growth and high neurogenic activity that is supported by radial glia progenitors. However, although synthesis of neuroprogestagens has been documented recently in the brain of zebrafish, information on the presence of progesterone receptors is very limited. In zebrafish, a single nuclear progesterone receptor (*pgr*) has been cloned and characterized. Here, we demonstrate that this *pgr* is widely distributed in all regions of the zebrafish brain. Interestingly, we show that Pgr is strongly expressed in radial glial cells and more weakly in neurons. Finally, we present evidence, based on quantitative PCR and immunohistochemistry, that nuclear progesterone receptor mRNA and proteins are upregulated by estrogens in the brain of adult zebrafish. These data document for the first time the finding that radial glial cells are preferential targets for peripheral progestagens and/or neuroprogestagens. Given the crucial roles of radial glial cells in adult neurogenesis, the potential effects of progestagens on their activity and the fate of daughter cells require thorough investigation.

## Introduction

In adult vertebrates, production of sex steroids has long been considered to be restricted to the gonads and, on a lower level, the adrenals. However, it is now acknowledged that *de novo* synthesis of steroids from cholesterol also occurs within the brain. The evidence is based on the fact that all steroidogenic enzymes are expressed in the brain and exhibit biological activity. The synthesis of such “neurosteroids” was initially demonstrated in mammals and subsequently in other vertebrates [1], [2], [3], [4], [5], [6], [7].

While many studies have focused on the feedback effects of sex steroids on the organization and regulation of neuroendocrine and behavioral circuits, there is increasing interest for non-reproductive functions of steroids in the brain, notably on neurogenesis and brain repair. This is particularly the case of estrogens and progesterone, whose effects on brain development and brain repair are more and more studied [8], [9]. In the case of progesterone and its metabolites, accumulating data indicate possible involvement in neurodevelopment and neuroprotection [10], [11], [12]. Neuroprotective effects of progesterone were notably demonstrated in experimental models of traumatic brain injury or ischemia [10], [11], [13], [14]. In addition, *in vitro* studies using brain slices of neonate rats showed that progesterone promotes dendritic growth and dendritic spine formation in Purkinje cells and that these effects are blocked by the progesterone receptor antagonist mifepristone [15], [16]. Recent studies in adult mice showed that progesterone enhances the survival and the migration of newborn neurons in the dentate gyrus [17] or in the subventricular zone (SVZ) of the hippocampus after a focal cerebral ischemia [18]. There is indication that neuroprotective effects of progesterone could be mediated by various mechanisms such as reduction of neuronal vulnerability to neurotoxic molecules [19], reduction of cell loss, inhibition of lipid peroxidation and expression of pro-inflammatory genes [11], [20].

In both the adult and developing rodent brain, multiple hypothalamic and extrahypothalamic sites are targeted by progesterone through activation of either nuclear or membrane receptors. In mammals, two nuclear progesterone receptor isoforms encoded by a single gene, PRA and PRB, mediate the genomic effects of progesterone [21]. More recently, it was shown that PRA and PRB can interact as dimers not only with DNA progesterone responsive element but also with signaling proteins of the Src/Ras/Erk pathway outside the nucleus [22], [23], [24]. The recent identification of membrane progesterone receptors adds further complexity to the picture [25], [26]. Interestingly, in rodents, studies have shown that various forebrain regions express nuclear receptors for progesterone (PRA and PRB) during specific periods of brain development. These include the preoptic-hypothalamic areas involved in reproductive neuroendocrine functions [27], but also regions not classically associated with reproduction, such as the thalamus, the hippocampus [27] and the neocortex [28], [29]. These data suggest that progesterone could also be involved in neural development.

The central effects of progesterone and/or its metabolites are poorly documented in teleost fishes. In fact, it is only known that 4-pregnen-17,20β-diol-3-one (17,20β-DHP) secreted by the ovary at the time of spawning acts as a pheromone stimulating sexual behavior [30], [31]. However, recent reports have established that the zebrafish brain exhibits 3-β-hydroxysteroid dehydrogenase/Δ-5-4 isomerase (3β-HSD) activity and expression [7], [32]. In addition, biochemical studies indicated that the progesterone synthetic pathway is very active, resulting in brain production of progesterone and progesterone derivatives, such as 17-hydroxyprogesterone and dihydro-progesterone. This suggests that, similar to the situation in mammals, progestagens actions in the fish brain result from both peripheral production and local synthesis.

Until now, virtually nothing is known regarding the expression of either nuclear or membrane-bound progesterone receptors in fish. The recent characterization of a unique nuclear progesterone receptor (*pgr*) in zebrafish and the development of antibodies to zebrafish Pgr now opens the possibility of investigating in detail the distribution of Pgr in the brain, and the phenotype of the cells expressing this Pgr [33], [34]. Nuclear PR receptor binding assays and dual transactivation/transcription assays clearly established that progestins, including progesterone, 17-hydroxyprogesterone, dihydro-progesterone and 17,20β-DHP specifically bind and activate zebrafish Pgr [33], [34]. Preliminary data indicated that Pgr is widely distributed in the brain of zebrafish [33], notably in the ventricular proliferative regions. In adult fish, the brain ventricles are lined by radial glial cells acting as neuronal progenitors and supporting the migration of newborn neurons [35], [36], [37], [38], [39], [40]. In contrast to mammals, in which radial glial cells become astrocytes at the end of embryogenesis [41], [42], [43], [44], [45], [46], radial glial cells persist in the whole brain during adulthood, supporting its constant growth. In the brain of adult zebrafish, radial glial cells express classical markers such as GFAP (Glial Fibrillary Acid Protein), BLBP (Brain Lipid Binding Protein), protein S100β, CXCR4 (a chemokine receptor) or nestin [35], [37], [38], [47], [48], [49]. In addition, radial glial cells in fish also express a number of steroidogenic enzymes, such as 3β-HSD [7] and aromatase B [7], [36], [38], [50], indicating that these cells potentially produce a wide variety of neurosteroids, notably estrogens and progesterone.

These data prompted us to investigate in more detail the expression and regulation of nuclear progesterone receptors in the brain of zebrafish. For this purpose, we performed *in situ* hybridization and immunohistochemistry using a specific zebrafish nuclear progesterone receptors riboprobe and antibody [33]. We then characterized the neuronal or radial glial nature of the Pgr expressing cells by performing double staining with acetylated-tubulin (neuronal marker) or by using the tg(*cyp19a1b*-GFP) transgenic zebrafish, which specifically expresses GFP in radial glial cells [38], [49], [51]. Finally, we examined the potential estrogenic regulation of nuclear progesterone receptors mRNA and protein in the brain of zebrafish.

## Materials and Methods

### Ethics

This study was approved by the ethics committee CREEA (Comité Rennais d'Ethique en matière d'Expérimentation Animale) permit number EEA B-35-040. All steps have been taken to reduce suffering of animals. Experiments were performed in accordance with European Union regulations concerning the protection of experimental animals (Directive 86/609/EEC).

### Animals

Experiments were performed on adult wild zebrafish (*Danio rerio*) and on transgenic tg(*cyp19a1b*-GFP) zebrafish. This line expresses GFP in radial glial cells under the control of *cyp19a1b* promoter [51]. Fishes were housed in the zebrafish facilities of the IFR140 (INRA SCRIBE, Rennes) and maintained under constant temperature (28.5°C) and photoperiod (14-hours light/10-hours dark). For sacrifice, fish were anesthetized on ice before spinal cord sectioning.

### *In situ* hybridization

Brains (n = 6) were partially dissected through skull opening and fish were immersed overnight at 4°C in 4% paraformaldehyde (PFA) in saline phosphate buffer (PBS, pH 7.4). On the next day, brains were taken out and fixed at 4°C in 4% PFA, in order to be processed for paraffin embedding and cut at 6 µm. Sections were mounted on poly-lysine slides for subsequent processing using the *in situ* hybridization protocol described previously by Diotel and colleagues [49]. Riboprobe synthesis was previously described by Hanna and colleagues [33]. In order to ascertain the *in situ* hybridization specificity, sense and antisense probes were always hybridized on parallel sets of slides.

### Immunohistochemistry

All staining was performed on frozen larvae sections or frozen brain sections (12 µm) from adult zebrafish (n = 6) prepared with a cryostat. Samples were fixed in 4% PFA overnight at 4°C. The samples were next immersed in PBS containing 30% of sucrose overnight, embedded in Tissue-tek before freezing at −80°C. Immunohistochemistry experiments were processed as previously described [33]. Briefly, tissue sections were first rinsed in PBS and non-specific binding was blocked for 1 hour at room temperature in PBS containing 0.2% triton and 0.5% milk powder. The antibody used was raised against peptides based on the N-terminal part (aa100–112) of zebrafish Pgr [33]. Sections were then incubated with rabbit anti-Pgr antibody (1:2500–1:5000) alone or with mouse anti-acetylated tubuline (1:100; Acetyl-Tub; clone 6-11B-1; Reference T 6793, Sigma) overnight at room temperature. After several washes in PBS 0.2% triton, tissue sections were incubated with the appropriate secondary antibodies (Goat anti-rabbit Alexa 594 or goat anti-mouse Alexa 488, Invitrogen) and slides were then rinsed in PBS-Triton. After immunohistochemical processing, cell nuclei were visualized with a DAPI counterstaining (Vectashield with DAPI, Vector Laboratories). The Pgr staining specificity has already been demonstrated [33]. Furthermore, omission of the primary or secondary antibodies caused no reaction.

### Zebrafish treatment and RNA extraction

Adult male zebrafish were exposed to either to 10^−7^ M of 17β-estradiol (17β-estradiol, Sigma-Aldrich, St. Louis, MO, USA) for 100 hours, or to 10^−6^ M of 1,4,6-androstatriene-3,17-dione (ATD) for 7 days, in glass tanks maintained at 28.5°C. Control fish were exposed to ethanol. Larvae were treated with 17β-estradiol (10^−8^ M in EtOH) or with EtOH alone, from 0 to 8 days post-fertilization. In both cases, water and hormones were replaced every day. After exposure, individual brains, pools of 5 brains of adult zebrafish, and pools of 20 larvaes were sonicated (10 sec, three times) in 1 mL Trizol Reagent (Gibco, Carlsbad, CA, USA), and total RNA was extracted according to the manufacturer's protocol. Each experiment was performed at least twice.

### Quantitative real-time PCR

Reverse transcription was carried out by incubating 2 µg total RNA with 1 µg of random primer oligonucleotides, 2.5 mM dNTPs and 50 U MMLV-RT (Promega) in the appropriate buffer 10 minutes at 65°C and 60 minutes at 37°C. Polymerase Chain Reactions (PCRs) were performed with an iCycler thermocycler coupled to the MyiQ detector (Bio-Rad. Hercules, CA, USA) using iQ SYBR-Green Supermix (Bio-Rad) according to the manufacturer's protocol. The following primers were used: EF-1 (fw) 5′-AGCAGCAGCTGAGGAGTGAT-3′; EF-1 (rev) 5′-CCGCATTTGTAGATCAGATGG-3′; *pgr* (fw) 5′-GAGCAATGATCAGCTGAGAAGG-3′; *pgr* (rev) 5′-TCCAGAGGAACAGTGTTGAGG-3′. Expression levels of EF-1 mRNA were used to normalize the expression levels between each sample. Triplicates of each sample were loaded in the 96 well-plate for each analyzed gene. Melting curve and PCR efficiency analyses were performed to confirm correct amplification. The Delta-Delta CT method was used for calculating the relative expression. The significance of fold induction was assessed using a Student's *t* test.

### Microscopy

Observations were carried out on an Olympus Provis photomicroscope equipped with a DP50 digital camera or with an epifluorescence Zeiss (Imager Z1, equipped with the Apotome module). Micrographs were taken in the TIFF format using the Analysis software, allowing image superposition. Images were then prepared with Adobe Photoshop CS4 for light or contrast adjustment before preparation of the plates.

### Pgr fluorescence intensity quantification

In order to quantify Pgr staining intensity in radial glial and parenchymal cells, pictures were converted into 8 bit. Using the ImageJ software, Pgr-positive cell nuclei located in the regions of interest were given a value according to a gray scale. In order to determine the effect of 17β-estradiol on Pgr fluorescence intensity, pictures of control and treated larvae were taken at the same exposure time. Numerous cells were then selected and the same method was used to analyze Pgr intensity. Significant differences were determined using a Student's *t* test. Three independent samples were analyzed.

### Nomenclature

The nomenclature for brain nuclei is according to that established in the zebrafish atlas by Wullimann and colleagues (1996) [52].

## Results

### Pgr expressing cells are largely distributed in the adult brain

The distribution of nuclear progesterone receptor mRNA and protein in the forebrain of adult zebrafish was assessed by *in situ* hybridization and immunohistochemistry. Overall, these two techniques yielded identical results and thus reciprocally validate each other in addition to the other controls

**Figure 1:**
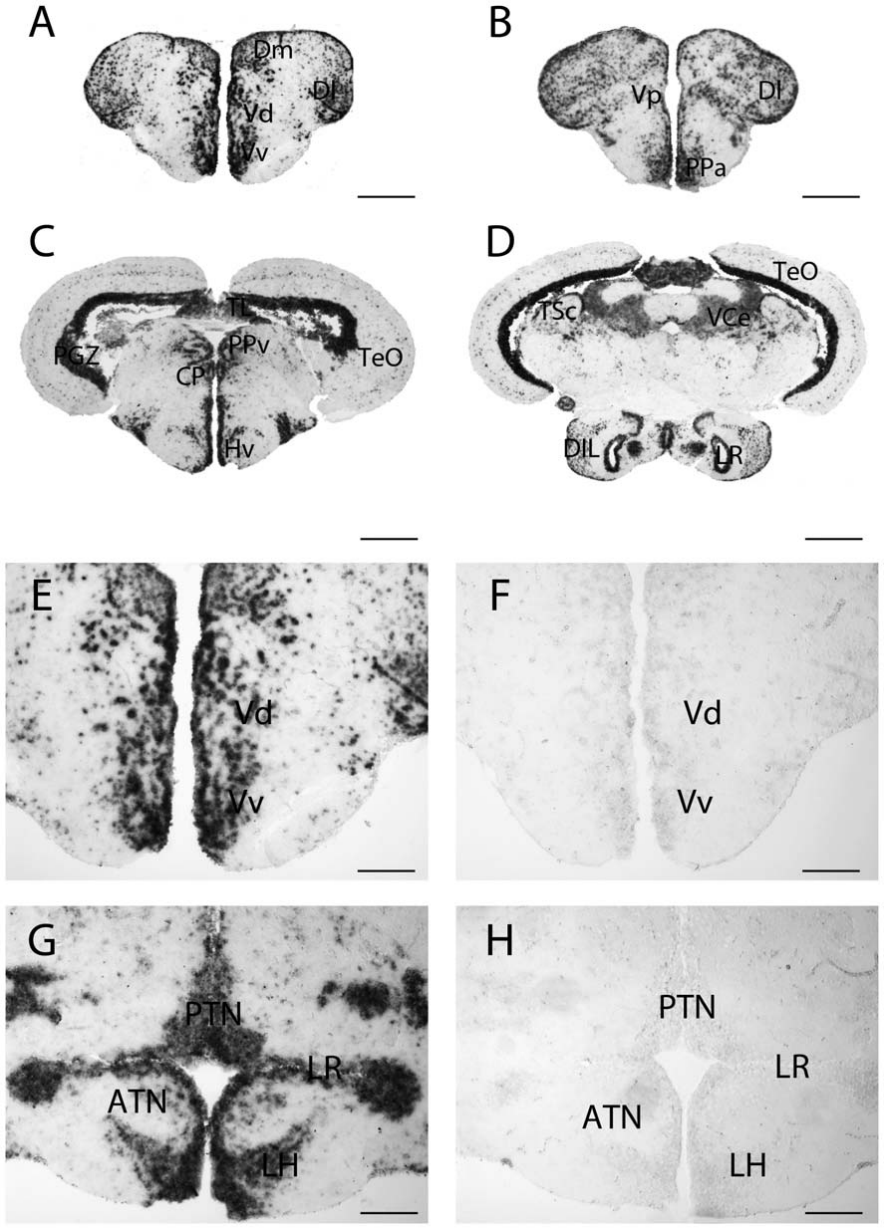
Expression of *pgr* transcripts in the forebrain of adult zebrafish. A, B, C and D: *pgr* transcripts detection with antisense probes in the telencephalon (A), the preoptic area (B), in the ventromedial hypothalamus (C) and the nucleus recessus posterioris (D). E, F, G and H: *In situ* hybridization with antisense probes (E and G) and sense probes (F and H) on parallel sections. The sense probe does not generate any signal. ATN: anterior tuberal nucleus; CP: central posterior thalamic nucleus; DIL: diffuse nucleus of the inferior lobe; Dl: Lateral zone of the dorsal telencephalic area; Dm: medial zone of the dorsal telencephalic area; Hv: ventral zone of the periventricular hypothalamus; LH: lateral hypothalamic nucleus; LR: Lateral recess; PTN: posterior tuberal nucleus; Vv: ventral nucleus of the ventral telencephalic area; Vd: dorsal nucleus of the ventral telencephalic area; PGZ: periventricular gray zone of the optic tectum; PPv: periventricular pretectal nucleus; TeO: Optic tectum; TL: torus longitudinalis; TSc: torus semicircularis; VCe: valvula cerebella. Scale bars: 50 µm (G and H); 100 µm (E and F); 200 µm (A and B); 400 µm (C and D).

Figure 1 shows transverse sections hybridized with the *pgr* anti-sense probe in the tel-, di- and mesencephalon (Figures 1A to 1D). In agreement with the RT-PCR data showing high *pgr* gene expression in the brain [33], *in situ* hybridization also revealed strong *pgr* mRNA expression, widely distributed in all brain regions. Figure 1 also presents parallel sections hybridized with the anti-sense and sense probes (Figures 1E-1H).

**Figure 2:**
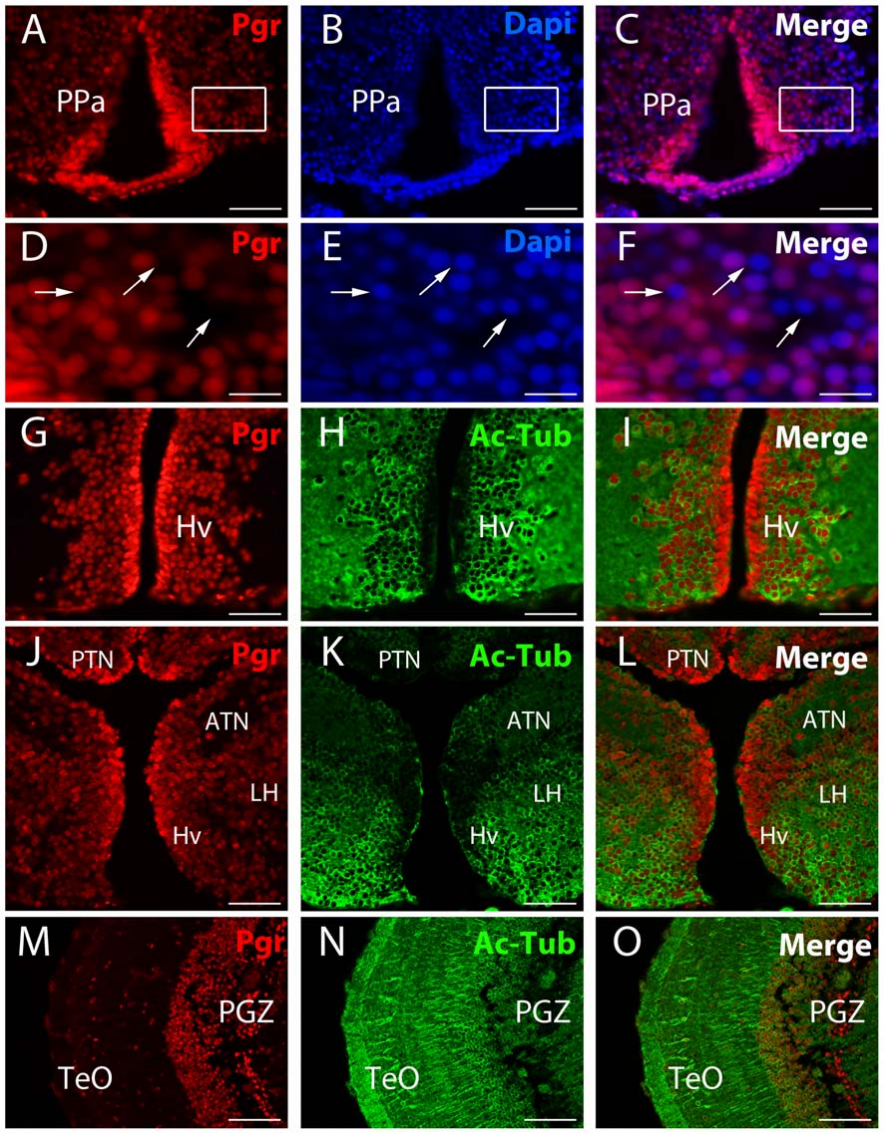
Immunolocalisation of *Pgr* expressing neurons in adult zebrafish forebrain. A to F: Pgr immunohistochemistry (red) with DAPI nuclei staining (blue) showing that not all nuclei express Pgr (arrows). D, E and F correspond to high magnifications of the area framed in A, B and C. Arrows point to nuclei that do not express Pgr. G to O: Pgr (red) and acetylated-tubulin (green) staining showing that numerous Pgr positive cells correspond to acetylated-tubulin positive neurons. Note that cells lining the ventricle are strongly positive for the Pgr antibodies, but are not stained by acetylated-tubulin. ATN: anterior tuberal nucleus; Hv: ventral zone of the periventricular hypothalamus; LH: lateral hypothalamic nucleus; PGZ: periventricular gray zone of the optic tectum; PPa: anterior part of the preoptic area; TeO: Optic tectum. Scale bars: 15 µm (D, E and F), 50 µm (A, B, C, G, H, I, J, K and L), 100 µm (M, N and O)

**Figure 3:**
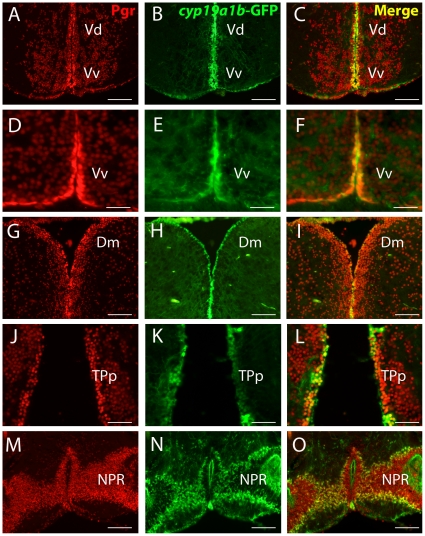
Radial glial cells strongly express *Pgr.* A to O: Pgr immunohistochemistry (red) on tg(*cyp19a1b*-GFP) transgenic fish showing the strong expression of Pgr in *cyp19a1b-GFP positive* radial glial cells (green). The *cyp19a1b*-GFP radial glial cells (green) that line the ventricular surface exhibit a stronger Pgr staining, notably in the subpallial (A to F) and pallial regions (G to I). This is also true in more posterior regions such as the posterior tuberculum (J to K) or around the posterior recess (NPR) of the caudal hypothalamus (M to O). Vv: ventral nucleus of the ventral telencephalic area; Vd: dorsal nucleus of the ventral telencephalic area; Dm: medial zone of the dorsal telencephalic area; TPp: periventricular nucleus of the posterior tuberculum; NPR: nucleus of the posterior recess of the hypothalamus Scale bars: 30 µm (D, E and F); 75 µm (J, K and L); 150 µm (A, B, C, G, H, I, M, N and O).

The *pgr* transcripts are found in numerous cells from the olfactory bulbs to the mesencephalon. Similarly, immunohistochemistry showed an identical widespread distribution of the Pgr protein (Figures 1, 2 and 3). Progesterone receptor expressing cells are distributed in all brain regions in many cells as shown by the DAPI counterstaining (Figures 2A–2F). However, DAPI counterstaining clearly indicated that a significant proportion of cells in every brain region do not express Pgr (Figures 2D–2F). Similar to the hybridization signal, a marked heterogeneity in the intensity of the immunohistochemical staining was also observed (Figures 2A–2F).

**Figure 4:**
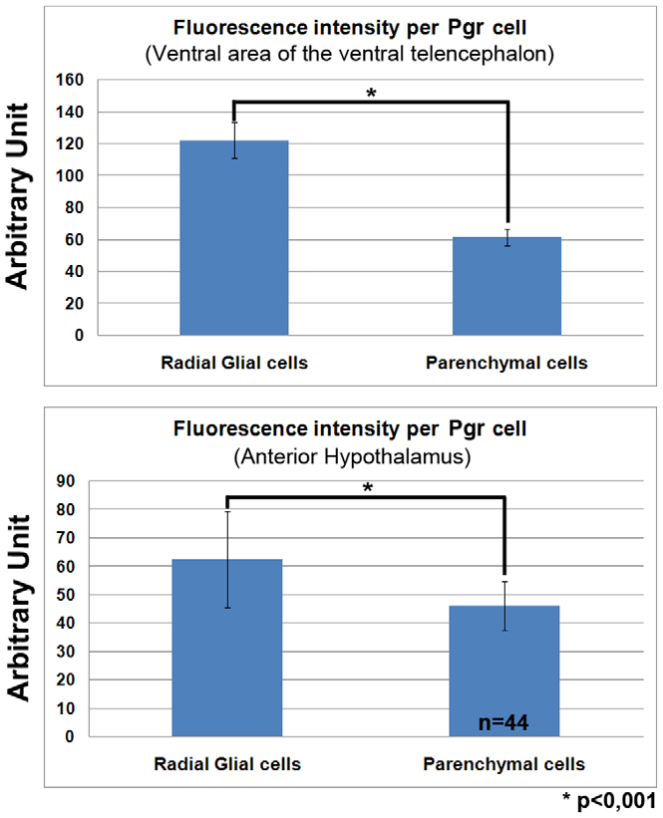
Expression of *Pgr* is stronger in radial glial cells than in neurons. Image analysis of the Pgr staining intensity in the ventral subpallium and in the anterior hypothalamus showing that radial glial cells exhibit a stronger Pgr immunoreactivity than parenchymal cells (p<0.001 Student's t test). The graphs present the mean value +/− the standard deviation.

The most anterior cells expressing Pgr were located in the granular cell layer of the olfactory bulbs (data not shown). Numerous labeled cells were detected in the telencephalic hemispheres (Figures 1A, 1E, 3A, 3D and 3G). Indeed, both *in situ* hybridization and immunohistochemistry generated intense labeling in both pallial and subpallial regions (Figures 1A, 1E, 3A, 3D and 3G). An obvious characteristic of the hybridization and immunohistochemical signals in the telencephalon was that the periventricular regions consistently exhibited stronger staining than cells in the parenchyma, which was also true at other brain levels (see below). For example, cells bordering the ventricles in the subpallial and pallial regions always expressed stronger Pgr staining compared to cells located in the parenchyma (Figures 3A, 3D and 3G). Quantification of Pgr staining by image analysis clearly showed that in the subpallium and the anterior hypothalamus, cells lining the ventricle exhibit significantly stronger staining than cells in the parenchyma (Figure 4).

Both *in situ* hybridization and immunohistochemistry produced very consistent staining in the anterior (PPa) and posterior part of the preoptic region and also in the post-commissural nucleus of the ventral telencephalic area (Vp) (Figure 1B, 2A and 2D). In the anterior preoptic region, Pgr protein and mRNA expressing cells were abundant in the parvocellular preoptic nucleus (PPa) (Figures 1B and 2A). Again, immunohistochemistry clearly showed that the ependymal layer was more heavily labeled than the parenchyma (Figures 2A and 4).

We also observed expression of nuclear progesterone receptors in the diencephalon notably in the ventromedial and ventrolateral thalamic nuclei (not shown), the ventral habenular nucleus, the periventricular nucleus of posterior tuberculum, and the diffuse nucleus of the inferior lobe (Figures 1D, 2J and 3J). Very high expression of *pgr* messengers and protein was also detected at all levels of the mediobasal hypothalamus (Figures 1C, 1G, 2G and 2J), in the periventricular regions of the lateral (Figure 1D and 1G) and posterior recesses (Figure 3M). Furthermore, we observed that cells surrounding the lateral recess (LR) strongly express *pgr* (Figure 1D and 1G). A remarkable finding was the very high expression of mRNA and protein in numerous cells of the posterior tuberal nucleus (PTN) and in cells lining the ventral zone of periventricular hypothalamus (Figures 1C, 1G, 2G and 2J). On the contrary, more laterally, in the lateral hypothalamic nucleus (LH) and in the anterior tuberal nucleus (ATN), the intensity of the Pgr labeling decreases while the high number of stained cells remains constant (Figure 2J).

We also observed Pgr expressing cells in the torus longitudinalis (Figure 1C) and in the optic tectum, notably in the densely packed small cells of the periventricular gray zone (PGZ) (Figure 1C, 1D and 2 M). The valvula cerebelli and the torus semicircularis also clearly expressed *pgr* (Figure 1D).

### Pgr is differentially expressed in neurons and radial glial cells

A major characteristic of the expression pattern of nuclear progesterone receptors, at both the mRNA and protein levels, was the consistently stronger signal identified along the brain ventricles, suggesting higher Pgr expression in radial glial cells than in surrounding neurons. In order to further characterize the Pgr expressing cells, we performed double staining experiment using a monoclonal antibody to acetylated tubulin, an established marker of neurons. We found that many cells located in the parenchyma exhibited a cytoplasm labeled by acetylated tubulin, but also a Pgr positive nucleus. As shown in Figure 2G–2O, this was observed in many regions. As expected, the cells bordering the ventricles did not contain acetylated-tubulin labeling (Figure 2G–2L). By performing Pgr immunohistochemistry on tg(*cyp19a1b*-GFP) transgenic fish, which express GFP in radial glial cells under control of the *cyp19a1b* promoter, we demonstrated that cells strongly expressing Pgr along the ventricles correspond to *cyp19a1b*-GFP positive radial glial cells (Figure 3A to 3O). Consequently, it appears that Pgr expression is higher in the estrogen-synthesizing radial glial cells.

### Nuclear progesterone receptor mRNA and protein are positively regulated by estrogens

**Figure 5:**
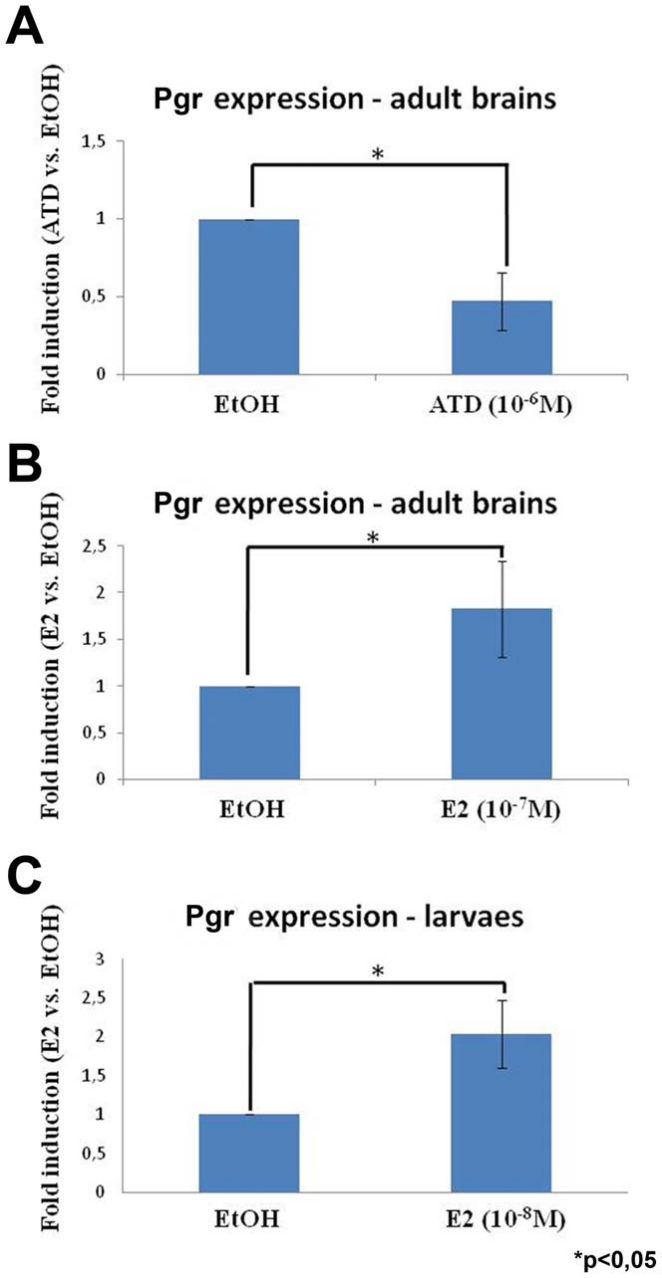
Nuclear progestin receptor is up-regulated by estrogen in both the developing and adult brain. Fold induction of *pgr* expression after treatment of adult zebrafish with an aromatase inhibitor (A, 10^−6^ M of ATD) or estradiol (B, 10^−7^ M of 17β-estradiol), and larvae with E2 (C, 10^−8^ M of 17β-estradiol). In A, the aromatase inhibitor (ATD) leads to a significant decrease of *pgr* expression in individual brains of adult zebrafish (n = 4). In B, the estrogenic treatment leads to a significant increase of *pgr* expression in pools of 5 brains of adult zebrafish (n = 3). In C, the estrogenic treatment leads to a significant increase of *pgr* expression in pool of 20 larvae (n = 2). Asterisk (*) indicates significant differences (p<0.05, Student's t test). The graphs present the mean value +/− the standard deviation.

Given that Pgr expression was higher in aromatase B expressing radial glial cells, we decided to investigate the potential involvement of the estrogenic environment on Pgr expression by treating fish either with an aromatase inhibitor (ATD) or with 17β-estradiol. The data generated by real time-qPCR clearly demonstrated that treatment with ATD (10^−6^ M) leads to a significant 50% decrease of *pgr* expression after 7 days of treatment (Figure 5A). In contrast, treatment of adult fish with 17β-estradiol (10^−7^ M) for 100 hours significantly increases *pgr* expression levels (Figure 5B).

**Figure 6:**
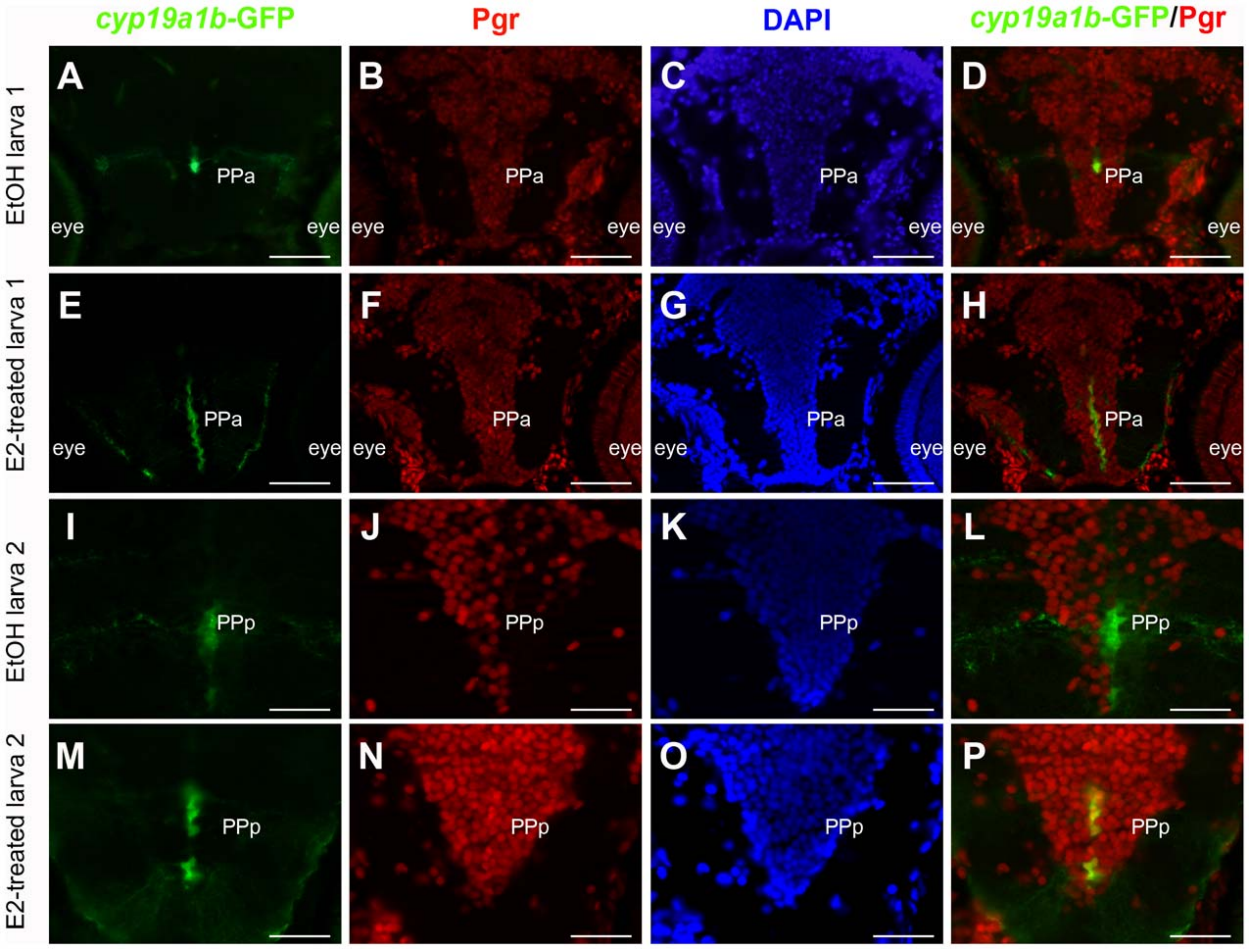
Nuclear progestin receptors are up-regulated by estradiol. A to P: Pgr immunohistochemistry (red) on tg(*cyp19a1b*-GFP) transgenic larvae (green) with DAPI counterstaining (blue) in the anterior (PPa) and posterior (PPp) preoptic area in two different control larvae (A–D and I–L) and two E2-treated larvae (E–H and N–P). A–D: Control larva treated with EtOH: Although the Pgr staining is widely distributed, not all cells are positive as shown by DAPI counterstaining. Only a few *cyp19a1b*-GFP expressing radial glial cells are visible and some of them express Pgr (D). E–H: Larva treated with 10^−8^ M of 17β-estradiol: At exactly the same level than in control (A–D), one can see that a majority of cells express Pgr as shown by the DAPI counterstaining. Furthermore, Pgr staining intensity is stronger than in controls. The estrogenic treatment also leads to an increase in the number of *cyp19a1b*-GFP positive radial glial cells expressing Pgr (H). Pictures in E and F were taken with the same exposure time than A and B, respectively. I–L: Control larva treated with EtOH shown at higher magnification in the posterior preoptic area. The Pgr staining is clearly detectable in some cells as indicated by the DAPI counterstaining. As shown by the merge picture in L, the *cyp19a1b*-GFP radial glial cells do not express Pgr. M–P: Larva treated with 10^−8^ M of 17β-estradiol (E2) in the exactly the same region than in I–L. Pgr is expressed in a large majority of cells and the staining intensity is clearly much stronger than in control larvae. Furthermore, all *cyp19a1b*-GFP positive radial glial cells also express Pgr. Pictures in M and N were taken with the same exposure time than I and J, respectively. Scale bars: 60 µm (A, B, C, D, E, F, G and H); 30 µm (I, J, K, L, M, N and O).

**Figure 7:**
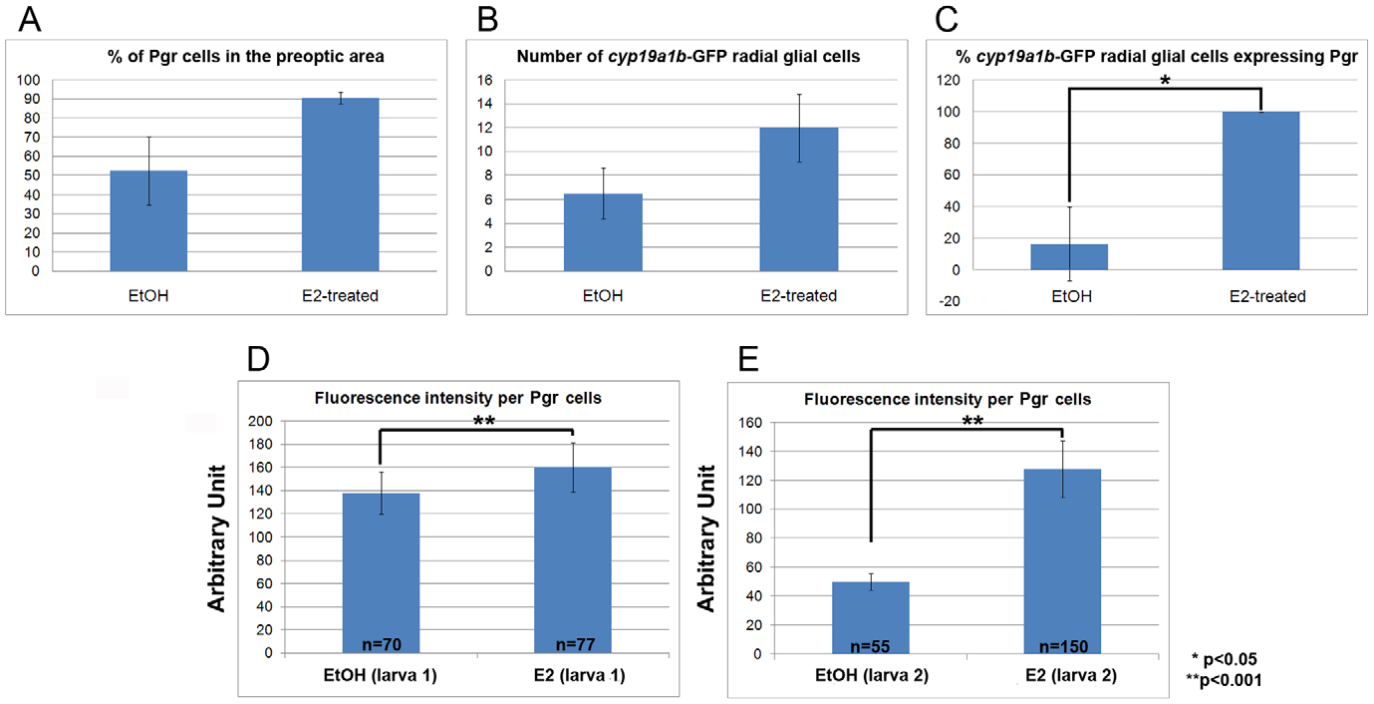
Effects of estradiol on *Pgr* expression in the preoptic area. The total number of Pgr, *cyp19a1b*-GFP and DAPI expressing cells were counted in the same region of the preoptic area in controls and E2-treated larvae, respectively (n = 3). A: Effect of estrogens on the proportion of DAPI positive cells expressing Pgr. B: Number of *cyp19a1b*-GFP expressing radial glial cells per section of control and E2-treated larva. C: Effect of estrogens on the proportion of *cyp19a1b*-GFP positive radial glial cells showing Pgr expression. D and E: Analysis of the fluorescence intensity of Pgr staining in control and estradiol-treated larvae shown in Figure 6 (D corresponds to Figures 6 B and F; E corresponds to Figures J and N). n  =  number of cells counted. The asterisk (*) means that the differences observed are significant (p<0.001, Student's t test). The graphs present the mean value +/− the standard deviation.

Treatment of eggs and larvae with 17β-estradiol (10^−8^ M) for 8 days also leads to a two fold increase in *pgr* mRNA expression as assessed by real time-qPCR (Figure 5C). This result was confirmed at the protein level using immunohistochemistry. Figure 6 shows that, as expected, E2 treatment increases *cyp19a1b*-GFP levels in radial glial cells and also increases the number of *cyp19a1b*-GFP positive radial glial cells [50]. In addition, 17β-estradiol causes increased expression of Pgr protein in radial glial cells along the brain ventricles, but also within neurons in the parenchyma (Figure 6). Although we observed that estradiol leads to an increase in the number of both parenchymal and *cyp19a1b*-GFP radial glial cells expressing Pgr, this increase was not significantly different (Figures 7 A and B). Furthermore, we noted that there were more *cyp19a1b*-GFP radial glial cells expressing Pgr in the E2-treated larvae compared to control larvae (Figure 7C). Finally, it appeared that the Pgr staining intensity was significantly stronger in the E2-treated larvae (Figures 7D and E). Such data suggest that estrogen treatment leads to an increase in Pgr expression, but also to a higher number of Pgr expressing cells corresponding to both neurons and radial glial cells.

## Discussion

The present study further documents the sites of Pgr expression in the brain of the zebrafish, a teleost fish. We clearly establish by both *in situ* hybridization and immunohistochemistry that the zebrafish brain is widely targeted by progesterone and/or progesterone derivatives, in particular in the preoptic area and the hypothalamus. However, we also show that Pgr is expressed in many cells of non-neuroendocrine regions. Additionally, we demonstrate that Pgr staining is stronger in radial glial cells than in neurons, implicating crucial progenitor cells as preferential targets of progesterone. Finally, we provide evidence that estrogens up-regulate Pgr expression in the brains of fish, similar to what is known in mammals.

### A wide expression of Pgr in the zebrafish brain

The localization and functions of Pgr in the fish brain are very poorly documented [33], [53]. The present study shows a very strong and widely distributed expression of *pgr* mRNAs in the whole brain, confirming the data obtained by immunohistochemistry. This further reinforces the specificity of the zebrafish Pgr antibody previously validated by western blotting [33]. In all cases, the staining is nuclear as expected for nuclear receptors. Apart from zebrafish, expression of Pgr in teleosts has only been documented in the African cichlid, *Astatotilapia burtoni*, in which similar results were obtained in terms of abundance and localization [53]. In mammals, *Pgr* is also widely expressed in the brain, notably in the preoptic area, the hypothalamus or also in the hippocampus [27], [54]. Interestingly, *Pgr* expression levels in rodents seem to be developmentally regulated [27]. In the developing forebrain of rat, numerous brain nuclei indeed appear to express *Pgr*, while, at postnatal day 28, expression is much lower in most brain regions [27]. In mammals, the phenotype of Pgr-expressing cells is partly documented. Nuclear progesterone receptors were notably found in dopaminergic neurons of the preoptic area and hypothalamus, but not in the ventral tegmental area [55]. In addition, Pgr in mammals were also observed in glial cells, including oligodendrocytes and astrocytes [8], [56], [57].

Despite this wide expression of Pgr in the brain of fish, there is virtually no data on the functions that could be potentially affected by progestagens.

### Pgr is strongly expressed in radial glial cells in adult zebrafish

A major finding of this study is the discovery that Pgr is not only detected in neurons, but that is also expressed in radial glial cells. Such cells were first described in the developing brain of mammals by Magini at the end of the 1800s [58]. By definition, they exhibit a small soma localized along the ventricles and extend two cytoplasmic processes, one to the ventricular surface and the other to the pial surface [58], [59]. In the developing brain of mammals, radial glia were first documented as a support system for the migration of newborn neurons [41], [44], [58], [60], [61], but it is now admitted that radial glial cells have more crucial functions. Indeed, they are acknowledged as neural stem cells that can give birth, directly or indirectly, to all cell types of the brain, including neurons, oligodendrocytes, ependymal cells and astrocytes [45], [46], [62]. In contrast to mammals in which radial glial cells transform into astrocytes at birth [42], [60], [62], [63], they persist in the brain of fish during adulthood.

Using the transgenic tg(*cyp19a1b*-GFP) zebrafish line that expresses GFP in radial glial cells [51], we unambiguously demonstrate Pgr expression in GFP expressing cells lining the ventricles. This result is particularly interesting in view of the fact that radial glial cells act as neural stem cells and sustain constant brain growth in adult fish [36], [37], [38], [64]. Recent data in zebrafish indicate that radial glial cells actively divide symmetrically or asymmetrically to generate both neurons and radial cells [36], [37], [40], [48]. These data have led some authors to suggest that the adult fish brain is a developing organ that conserves some features of the embryonic mammalian brain [36], [38], [39], [40], [65]. This could explain the abundance of Pgr in the brain of fish. Indeed, it was recently proposed that the transient expression of *Pgr* in many brain regions during development in rodents reflects a role for progesterone and Pgr in fundamental mechanisms of neural development [27]. During brain development in rats, the nuclear progesterone receptor is transiently expressed in regions not typically associated with neuroendocrine functions, such as the neocortex, the dentate gyrus, the caudato-putamen, the periventricular striatum [27]. This suggests a developmental function for progesterone in these regions and reinforces the view that steroid hormones could play a significant role in neurogenesis [8], [9], [27], [38], [64]. Studies in rats also suggest that Pgr expressed in the subventricular zone participate in neural stem cell proliferation [27]. Whether progesterone is involved in radial glial cell proliferation in developing or adult fish will have to be investigated. In any case, this raises the question of the source, peripheral or central, of progesterone available for Pgr binding during embryonic brain development in mammals or adult neurogenesis in fish.

### Radial glial cells: Source and target for neuroprogesterone

Our own results in zebrafish suggest that, the brain, in addition to the gonads, is a source of neurosteroids [7], [32]. Similar to other vertebrates [1], [2], [3], [4], [5], [6], the brain of fish is able to produce a number of steroids, including progesterone and progesterone derivatives, suggesting a role for progestagens in brain functions [7], [32]. In particular, the steroidogenic enzyme 3β-HSD which catalyzes the oxidation and isomerization of pregnenolone into progesterone is expressed in the brain of zebrafish. In zebrafish, 3β-HSD activity was first reported in the adult brain by Sakamoto and colleagues [32]. Our recent experiments confirmed the presence of 3β-HSD activity and expression in the brain of adult zebrafish, which also expresses other steroidogenic enzymes (Cyp11a1, Cyp17; 17β-Hsd, 3α-Hsd, 5α-reductase and aromatase) [7]. Interestingly, it also appears that radial glial cells could express *3β-hsd, cyp11a1* and *cyp17* mRNAs in addition to the estrogen synthesizing enzyme aromatase [36], [50]. Thus, the brain of adult zebrafish appears to be a steroidogenic organ, able to synthesize its own (neuro)steroids which act locally to regulate some brain functions, potentially neurogenesis and plasticity [7].

In mammals, astrocytes are also known to be both a source of neurosteroids, including progesterone [66], [67], [68], [69], [70], and a target for steroids and/or neurosteroids [3], [6], [56], [57], [71]. Until now, there is no solid evidence that the forebrain of fish contains *bona fide* star shaped astrocytes. This is understandable in view of the fact that radial glial cells persist throughout life in fish while they transform into astrocytes at birth in mammals [72], [73]. Furthermore, radial glial cells in adult fish express many of the canonical markers of the astrocyte lineage in mammals [35], [36], [37], [48], [49], [74]. Thus, all these data suggest that radial glial cells in zebrafish are both a source and a target for neuroprogesterone.

### Estrogens up-regulate nuclear progesterone receptor expression

While it is well known that *Pgr* is up-regulated by estrogens in the brain of mammals, our data are the first to demonstrate that nuclear progesterone receptors are also up-regulated by estrogens in the brain of zebrafish at both the mRNA and the protein levels. Evidence based on qPCR experiments shows that fish treated with ATD, an aromatase inhibitor, exhibit lower brain *pgr* expression, whereas fish treated with estrogens exhibit a significant increase in *pgr* mRNA levels. Experiments in larvae and adult brains further reinforced this result by showing that E2 increased *pgr* mRNAs (q-PCR) and Pgr immunoreactivity as measured by image analyses. Such data demonstrate that estradiol treatment significantly increased progesterone receptor expression [8], [67], [68], [69], [75], [76]. According to our immunohistochemical results, the *pgr* up-regulation upon E2 treatment would concern both neurons and radial glial cells. In the brain of teleosts, there is evidence for the presence of nuclear estrogen receptor esr1, esr2a and esr2b in many brain regions, in particular in the periventricular regions of the forebrain, in neurons and probably in radial glial cells [77], [78], [79] (N. Diotel et. al, unpublished).

### Conclusions

Complementing our previous studies on the expression of steroidogenic enzymes in the brain of zebrafish [7], [38], the present report provides for the first time detailed information on the distribution and nature of Pgr positive cells. The data indicate a widespread localization of Pgr in many brain regions, suggesting actions outside the reproductive sphere. This receptor is present in many neurons throughout the brain, but it is obvious that radial glial cells exhibit stronger Pgr staining. Such data are particularly significant given the fact that radial glial cells are now recognized as progenitors sustaining brain growth in adult fishes. Additionally, such cells are also unique in strongly expressing brain aromatase and, to a lesser extent, other steroidogenic enzymes. Radial progenitors thus appear to be both sources and targets of neurosteroids, notably progestagens. In addition, we also show that, similar to mammals, *pgr* expression is up-regulated by E2. Altogether, these results suggest that the potential functions of progestagens, of peripheral and central origins, have been overlooked until now and should be investigated in particular in relation to the neurogenic activity of radial progenitors under physiological conditions, but also under situations of brain repair.
